# Genotoxicological Characterization of (±)cis-4,4′-DMAR and (±)trans-4,4′-DMAR and Their Association

**DOI:** 10.3390/ijms23105849

**Published:** 2022-05-23

**Authors:** Monia Lenzi, Sofia Gasperini, Veronica Cocchi, Micaela Tirri, Matteo Marti, Patrizia Hrelia

**Affiliations:** 1 Department of Pharmacy and Biotechnology, Alma Mater Studiorum University of Bologna, 40126 Bologna, Italy; m.lenzi@unibo.it (M.L.); patrizia.hrelia@unibo.it (P.H.); 2 LTTA Center and University Center of Gender Medicine, Department of Translational Medicine, Section of Legal Medicine, University of Ferrara, 44121 Ferrara, Italy; micaela.tirri@unife.it (M.T.); matteo.marti@unife.it (M.M.); 3 Collaborative Center for the Italian National Early Warning System, Department of Anti-Drug Policies, Presidency of the Council of Ministers, 00186 Rome, Italy

**Keywords:** (±)cis-4,4′-DMAR, (±)trans-4,4′-DMAR, novel psychoactive substances, stimulant, genotoxicity, In Vitro Mammalian Cell Micronucleus Test, flow cytometry

## Abstract

The novel psychoactive substance (NPS) 4-Methyl-5-(4-methylphenyl)-4,5-dihydroxazol-2-amine (4,4′-DMAR) shows psychostimulant activity. Data on the acute toxicity of 4,4′-DMAR are becoming increasingly available, yet the long-term effects are still almost unknown. In particular, no data on genotoxicity are available. Therefore, the aim of the present study was to evaluate its genotoxic potential using the “In Vitro Mammalian Cell Micronucleus Test” (MNvit) on (±)cis-4,4′-DMAR and (±)trans-4,4′-DMAR and their associations. The analyses were conducted in vitro on human TK6 cells. To select suitable concentrations for MNvit, we preliminarily evaluated cytotoxicity and apoptosis. All endpoints were analysed by flow cytometry. The results reveal the two racemates’ opposite behaviours: (±)cis-4,4′-DMAR shows a statistically significant increase in micronuclei (MNi) frequency that (±)trans-4,4′-DMAR is completely incapable of. This contrast confirms the well-known possibility of observing opposite biological effects of the cis- and trans- isomers of a compound, and it highlights the importance of testing single NPSs that show even small differences in structure or conformation. The genotoxic capacity demonstrated stresses an additional alarming toxicological concern related to this NPS. Moreover, the co-treatments indicate that consuming both racemates will magnify the genotoxic effect, an aspect to consider given the unpredictability of illicit drug composition.

## 1. Introduction

Since 2008, the worldwide use of synthetic stimulants has increased and now represents the largest group of novel psychoactive substances (NPSs) trafficked in the illicit drug market [1,2]. Indeed, synthetic stimulants seem to be increasingly popular among young adults (age 15–35) and have quickly established themselves as a public health emergency [2]. Well-known for their ability to replicate the effects of traditional psychostimulant drugs (such as cocaine, MDMA and amphetamines), synthetic stimulants have gained popularity for their enhanced power and low cost, representing the second largest group of NPSs monitored by the European Monitoring Centre for Drugs and Drug Addiction (EMCDDA) [2].

An NPS with psychostimulant activity, 4-Methyl-5-(4-methylphenyl)-4,5-dihydroxazol-2-amine (4,4′-DMAR) was first recognized in the Netherlands in November 2012 [3,4]. Within a span of only 9 months (between June 2013 and February 2014), this central nervous system (CNS) stimulant was linked to thirty-one deaths and one non-fatal intoxication [5]. In February 2014, following its rapid spread in the illicit market and evidence of intoxications and fatalities associated with this molecule, the EMCCDA and Europol produced a Risk Assessment Report on 4,4′-DMAR [3,6].

The NPS 4,4′-DMAR is available on the Internet, typically as a white powder or as tablets of different colours, shapes and sizes, and it can be sold under various street names, such as “Serotoni”, “Speckled Cherry”, “Speckled Cross” or “4-methyl-euphoria” [6,7].

Both tablets and powders contain 4,4′-DMAR in different dosages, alone or combined with other psychoactive substances, including cocaine, amphetamines, 3,4-methylenedioxymethamphetamine (MDMA) and synthetic cathinones [3,8]. 

The main routes of administration reported for 4,4′-DMAR are nasal insufflation, oral administration and inhalation [9].

In online discussions, anonymous drug users reported the dose ranges to achieve various desired effects, both psychological and behavioural, such as euphoria, talkativeness, increased sociability, physical energy and mental stimulation [10]. However, there is also a wide range of potential negative effects, including nausea, agitation, hyperthermia, convulsions and increased heart rate, which could result in cardiac arrest [6].

Despite the fact that 4,4′-DMAR is a widely sold synthetic stimulant with the above-mentioned online forums confirming its use and popularity, there is still much we do not know about this substance [8].

Chemically, 4,4′-DMAR is a substituted oxazoline that derives from the stimulants 2-amino-5-phenyl-2-oxazoline (aminorex) and 4-methylaminorex (4-MAR) [9,11,12]. The only structural difference between these three stimulants is the presence or absence of methyl groups [9]. In particular, aminorex has no methyl groups, 4-MAR only one, and 4,4′-DMAR has two methyl groups: one in position 4 of the oxazoline ring (also present in 4-MAR) and one in para-position on the phenyl ring. Consequently, 4,4′-DMAR, like 4-MAR, has two chiral centres that produce two different (±)cis and (±)trans racemates [5,7].

Concerning pharmacodynamics, in vitro studies have shown that 4,4′-DMAR acts on the monoamine system [4,12,13]. In particular, both diastereomeric forms of 4,4′-DMAR have proven to be equally effective in inducing the release of dopamine (DA) and noradrenaline (NA). The substantial difference between the two racemates concerns their serotonergic activity: (±)cis-4,4′-DMAR shows a strong stimulating action on the serotonin transporter (SERT), while (±)trans-4,4′-DMAR exhibits partial action [13]. Furthermore, Maier et al. demonstrated the ability of (±)cis-4,4′-DMAR to inhibit rat and human isoforms of vesicular monoamine transporter 2 (VMAT-2), suggesting a possible long-term neurotoxicity. Therefore, 4,4′-DMAR acts as a non-selective monoamine-releasing agent [14].

More recently, in a mouse in vivo study, the two DMAR stereoisomeric forms also demonstrated opposite toxicity: (±)trans-4,4′-DMAR did not show toxic effects; conversely, (±)cis-4,4′-DMAR exhibited severe levels of toxicity, including salivation, convulsions, hyperthermia and death, attributable to the serotonin syndrome. Critical data obtained following the co-administration of the two DMAR stereoisomers showed a worsening of the toxic effects caused by (±)cis-4,4′-DMAR, resulting in rapid and severe hyperthermia, convulsions and death [15].

These emerging data suggest a clearly acute toxicity profile of 4,4′-DMAR. Nevertheless, the long-term effects, including genotoxicity, whose role in developing several degenerative diseases is widely recognized, remain almost unknown. It is becoming increasingly urgent to understand these long-term effects for the sake of improving public health worldwide.

For this reason, the aim of the present in vitro study is to evaluate the genotoxic potential of (±)cis-4,4′DMAR and (±)trans-4,4′-DMAR and their co-treatment in human lymphoblastoid TK6 cells, particularly in terms of their ability to induce structural and numerical chromosomal aberrations using the “In Vitro Mammalian Cell Micronucleus Test” (MNvit), correspondent to OECD guideline n° 487 [16]. To define the concentrations to be used in the micronuclei (MNi) frequency evaluation, we proceeded according to the following experimental scheme: in the first phase of the research, we selected the concentrations at which cytotoxicity and cytostasis were absent or below the maximum level allowed by the OECD. Afterwards, we checked that the apoptosis of cultures treated with these concentrations was comparable to that measured in the untreated control cultures.

Once we had defined the concentrations to be tested, we evaluated the MNi frequency by a flow cytometric (FCM) protocol developed in our laboratory and subsequently published [17].

## 2. Results

### 2.1. Cytotoxicity Evaluation

**Figure 1:**
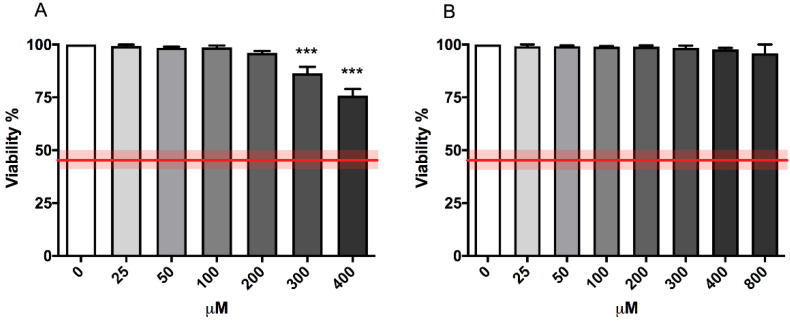
Cell viability of TK6 cells after 26 h treatment with (±)cis-4,4′-DMAR (**A**) or (±)trans-4,4′-DMAR (**B**) at the indicated concentrations compared to the concurrent negative control [0 µM]. Each bar represents the mean ± SEM of three independent experiments. Data were analysed using one-way repeated measures ANOVA followed by Dunnet as post-test. *** *p* < 0.001 vs. [0 µM]. The red line represents the OECD threshold for viability (45 ± 5%).

OECD guideline n°487 established a cytotoxicity threshold equal to 55 ± 5% to select the top concentration to be tested for the MNi frequency evaluation, and it consequently recommends proceeding only if the treated populations show a cell viability and a cell proliferation of at least 45 ± 5% when compared to concurrent negative control cultures [16]. Figure 1 shows the compliance of the cytotoxicity induced by both 4,4′-DMAR forms with the OECD guideline threshold (represented by the red line) at all the concentrations tested. However, (±)cis-4,4′-DMAR has proven to be slightly more cytotoxic in a concentration-dependent way compared to (±)trans-4,4′-DMAR.

Meanwhile, the correct cell replication, calculated as Relative Population Doubling (RPD), was also checked according to the formula reported in Materials and Methods Section 4.4.1 [16].

RPDs resulted above the OECD guideline threshold (equal to 45 ± 5%) up to the concentration 300 µM for (±)cis-4,4′-DMAR and at all the concentrations tested for (±)trans-4,4′-DMAR (Table 1).

Also in this case, the different cytostatic effects exerted by the two compounds is appreciable and for (±)cis-4,4′-DMAR is concentration-dependent (Table 1).

### 2.2. Apoptosis Evaluation

This test was performed to analyse a cell-death mechanism alternative to necrosis and to check that the apoptosis induction in treated cultures was similar or corresponding at most to twice the value of that recorded in the concurrent negative cultures. In particular, the three highest concentrations that respected the OECD cytotoxicity thresholds were evaluated for each substance: 100, 200 and 300 µM for (±)cis-4,4′-DMAR and 300, 400 and 800 µM for (±)trans-4,4′-DMAR. Etoposide (ETP) at 5µg/mL was used as positive control.

**Figure 2:**
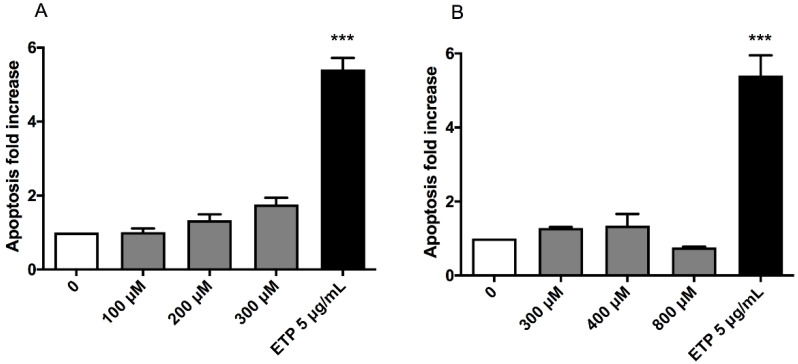
Apoptosis fold increase in TK6 cells after 26 h treatment with (±)cis-4,4′-DMAR (**A**), (±)trans-4,4′-DMAR (**B**) or the positive control ETP at the indicated concentrations compared to the concurrent negative control [0 µM]. Each bar represents the mean ± SEM of three independent experiments. Data were analysed using one-way repeated measures ANOVA followed by Bonferroni as post-test. *** *p* < 0.001 vs. [0 µM].

Apoptosis never doubled compared to that measured in the concurrent negative control for both compounds, in fact, it can only be noticed an apparent but not statistically significant apoptosis fold increase for (±)cis-4,4′-DMAR (Figure 2). 

### 2.3. MNi Frequency Evaluation

Based on the results obtained from viability, RPD and apoptosis, three concentrations to be tested in the MNi frequency evaluation were selected for each compound: 100, 200 and 300 µM for (±)cis-4,4′-DMAR and 300, 400 and 800 µM for (±)trans-4,4′-DMAR.

To assess the potential MNi induction by (±)cis-4,4′-DMAR and (±)trans-4,4′-DMAR, the number of MNi was evaluated in untreated control cultures, 4,4′-DMAR-treated cultures and positive-controls-treated cultures, i.e., treated with Mytomicin C (MMC) or Vinblastine (VINB).

**Figure 3:**
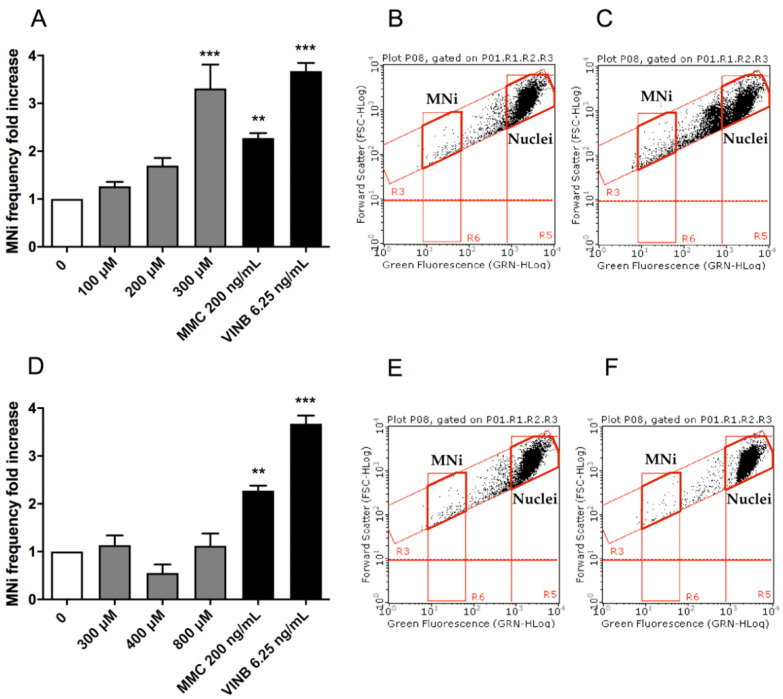
MNi fold increase in TK6 cells after 26 h treatment with (±)cis-4,4′-DMAR (**A**) and (±)trans-4,4′-DMAR (**D**) or positive controls (MMC, VINB) at the indicated concentrations compared to the concurrent negative control [0 µM]. Each bar represents the mean ± SEM of three independent experiments. Data were analysed using one-way repeated measures ANOVA followed by Dunnet as post-test. ** *p* < 0.01 vs. [0 µM]; *** *p* < 0.001 vs. [0 µM]. Dot plots obtained by FCM in the concurrent negative controls (**B**,**E**), (±)cis-4,4′-DMAR 300 µM (**C**) and (±)trans-4,4′-DMAR 800 µM (**F**). “MNi” and “Nuclei” indicate the regions of the dot plot where MNi and nuclei can be found.

Also in this analysis, the two test substances showed different effects: (±)cis-4,4′-DMAR caused a statistically significant increase in the MNi frequency fold increase, more than tripling, at 300 µM (Figure 3A), while a visible but not statistically significant increase was observed at 200 µM. On the other hand, (±)trans-4,4′-DMAR was associated with an MNi frequency fold increase consistently comparable to that measured in the negative control cultures at all the concentrations tested (Figure 3D). Representative FCM dot plots are reported in Figure 3 to support the results obtained. In particular, they illustrate how MNi are much more numerous in (±)cis-4,4′-DMAR-treated cultures (Figure 3C) than in the concurrent negative control cultures (Figure 3B), while in (±)trans-4,4′-DMAR-treated cultures (Figure 3F), they are comparable to the concurrent negative control cultures (Figure 3E).

Considering these results, which demonstrate the different behaviours of the two racemates, the associations between the two were tested as well. In particular, cell cultures were co-treated with the highest concentration of (±)trans-4,4′-DMAR, which showed no genotoxic effect, i.e., 800 µM, and the different concentrations of (±)cis-4,4′-DMAR, i.e., 100, 200 and 300 µM.

**Figure 4:**
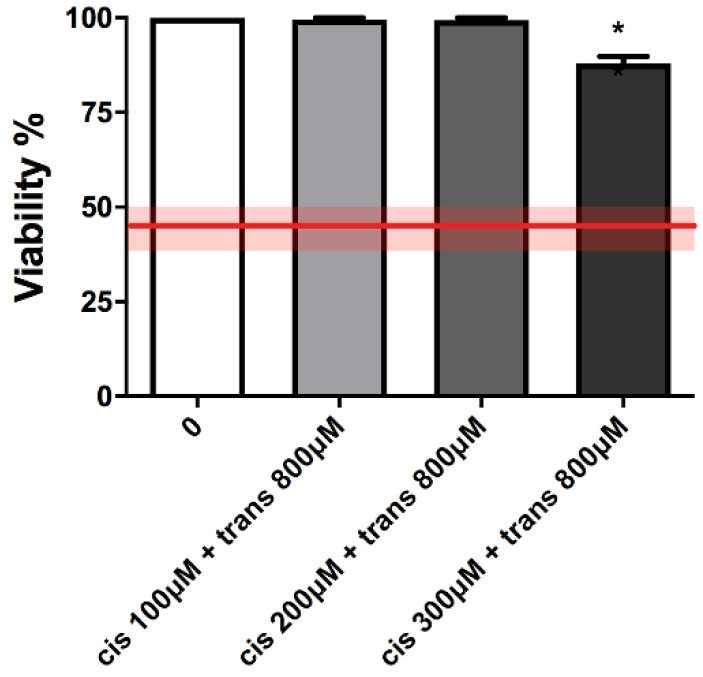
Cell viability of TK6 cells after 26 h co-treatment with (±)cis-4,4′-DMAR and (±)trans-4,4′-DMAR at the indicated concentrations compared to the concurrent negative control [0 µM]. Each bar represents the mean ± SEM of three independent experiments. Data were analysed using one-way repeated measures ANOVA followed by Bonferroni as post-test. * *p* < 0.05 vs. [0 µM]. The red line represents the OECD threshold for viability (45 ± 5%).

Again in this case, cytotoxicity and apoptosis were checked before proceeding with the MNi frequency evaluation. As shown in Figure 4 and Table 2, cell viability and RPDs remained within OECD limits (45 ± 5%).

**Figure 5:**
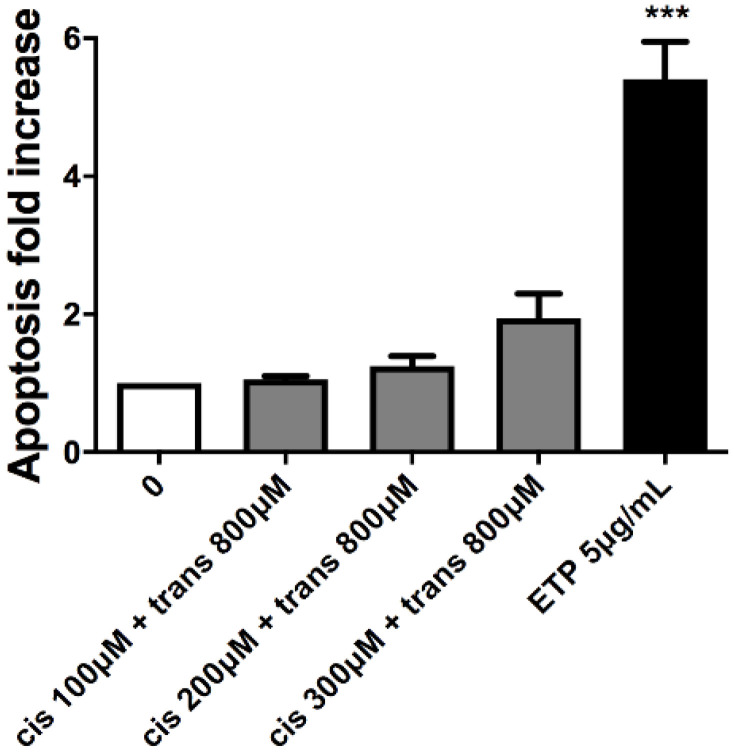
Apoptosis fold increase in TK6 cells after 26 h co-treatment with (±)cis-4,4′-DMAR and (±)trans-4,4′-DMAR or the positive control ETP at the indicated concentrations compared to the concurrent negative control [0 µM]. Each bar represents the mean ± SEM of three independent experiments. Data were analysed using one-way repeated measures ANOVA followed by Bonferroni as post-test. *** *p* < 0.001 vs. [0 µM].

Moreover, the apoptosis fold increase never doubled in value with any of the co-treatments tested compared to the concurrent negative control culture (Figure 5).

Overall, the results obtained allowed us to proceed with the MNi frequency evaluation, which demonstrated that the association with the non-genotoxic concentration 800 µM of (±)trans-4,4′-DMAR aggravates the effect of (±)cis-4,4′-DMAR. In particular, the MNi frequency fold increase in the cultures co-treated with (±)cis-4,4′-DMAR 200 µM was nearly twice as much as that recorded in the cultures treated with (±)cis-4,4′-DMAR 200 µM alone (2.91 vs. 1.70, respectively), resulting in a statistically significant increase.

Analogously, the association with (±)cis-4,4′-DMAR 300 µM also determined a statistically significant increase, greater than that of (±)cis-4,4′-DMAR 300 µM alone (4.43 vs. 3.31, respectively).

**Figure 6:**
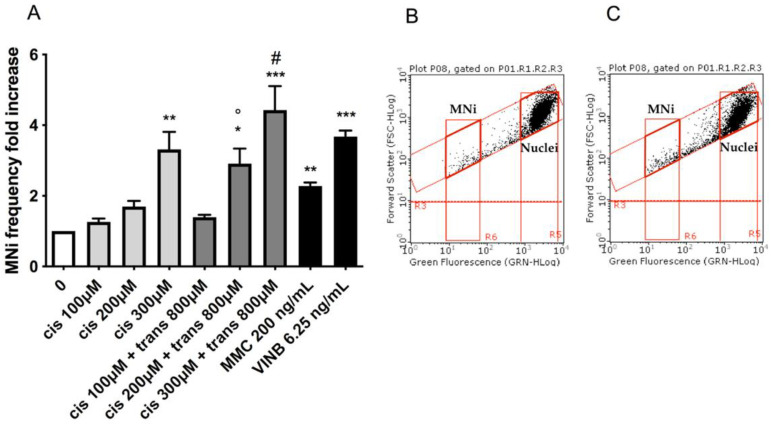
MNi fold increase in TK6 cells after 26 h treatment with (±)cis-4,4′-DMAR, (±)trans-4,4′-DMAR, their associations or positive controls (MMC, VINB) at the indicated concentrations compared to the concurrent negative control [0 µM] (**A**). Each bar represents the mean ± SEM of three independent experiments. Data were analysed using one-way repeated measures ANOVA followed by Dunnet as post-test. * *p* < 0.05 vs. [0 µM]; ** *p* < 0.01 vs. [0 µM]; *** *p* < 0.001 vs. [0 µM]. ° *p* < 0.05 vs. [cis 200 µM]. # *p* < 0.05 vs. [cis 300 µM]. Dot plots obtained by FCM in cis 200 µM (**B**) and cis 200 µM + trans 800 µM (**C**). “MNi” and “Nuclei” indicate the regions of the dot plot where MNi and nuclei can be found.

Conversely, (±)cis-4,4′-DMAR 100 µM and its association with (±)trans-4,4′-DMAR 800 µM were comparable (1.27 vs. 1.40, respectively) (Figure 6).

## 3. Discussion

The available toxicological data regarding the two stereoisomeric forms of 4,4′-DMAR, (±)cis-4,4′-DMAR and (±)trans-4,4′-DMAR, are extremely limited, and genotoxicological data are totally absent. Therefore, in this work, we investigated this aspect with particular attention to the possible difference between the assumption of the single isomer or the co-assumption of both in a variable mixture.

To assess the genotoxicity, the first fundamental step was to define the concentrations of the test chemical to be tested, which must lead to absent or poor cell death and no or limited inhibition of cell proliferation [16]. For this reason, OECD guideline n°487 established a cytotoxicity threshold equal to 55 ± 5% and consequently recommends proceeding only if the treated population shows a cell viability and a cell proliferation of at least 45 ± 5% when compared to concurrent negative control cultures [16].

For this purpose, we performed a cytotoxicity assay to evaluate the cell viability of untreated cultures and cultures treated with (±)cis-4,4′-DMAR scalar concentrations from 0 to 400 µM or (±)trans-4,4′-DMAR from 0 to 800 µM. The results obtained show that both (±)cis-4,4′-DMAR and (±)trans-4,4′-DMAR-treated cultures complied with the threshold at all the concentrations tested, but they also highlight a slightly more cytotoxic effect for (±)cis-4,4′-DMAR.

The same assay also allowed us to carry out a robust measurement of the number of cells at the time of cellular seeding (time zero) and at the end of the treatment time, which is fundamental in order to have a highly accurate understanding of the correct cell proliferation. Also in this case, the two 4,4′-DMAR forms showed different effects: the threshold was respected only up to 300 µM for (±)cis-4,4′-DMAR and at all the concentrations tested for (±)trans-4,4′-DMAR. Thus, (±)cis-4,4′-DMAR was more cytotoxic, both in terms of reduction in cell viability and reduction in cell proliferation.

The limited cytotoxicity could be considered as a positive result, but this is not entirely true from a genotoxicological point of view. Indeed, a substance capable of damaging DNA but allowing the cellular population to survive and replicate also means it is able to transmit any genetic damage to its offspring.

The cytotoxicity assay enables an effective discrimination between viable and necrotic cells, but it does not highlight apoptotic cells. In fact, the distinction is based solely on the difference in membrane integrity and the consequent permeability to the dye used. For this reason, apoptotic cells, characterized by a still-intact membrane, could be “mistaken” for living cells by the instrument. Therefore, we considered it necessary to proceed with a more specific test to highlight this alternative death mechanism. We performed the apoptosis assay to evaluate the apoptosis levels in cultures not treated and treated with (±)cis-4,4′-DMAR 100, 200 and 300 µM or (±)trans-4,4′-DMAR 300, 400 and 800 µM, concentrations selected based on the results obtained in the previous cytotoxicity assays. The double staining 7-AAD/Annexin V-PE verified that apoptosis never as much as doubled compared to that measured in the concurrent negative control in either compound. This aspect is particularly important in the genotoxicity evaluation since the cell population exposed to a genotoxic agent could be stimulated to undergo apoptosis following unrepaired genetic damage. On the contrary, resistance to apoptosis can result in the inability of cells to counteract, through this mechanism of selective death, the transmission of genetic damage to the daughter cells. Also at this toxicological endpoint, the two racemates of 4,4′-DMAR showed different behaviours. In fact, the increase in apoptosis levels, albeit slight, was greater for (±)cis-4,4′-DMAR than for (±)trans-4,4′-DMAR. 

OECD guideline n °487 lists different MNi scoring procedures, of which we selected FCM because it offers numerous advantages compared to optical microscopy, such as greater objectivity and statistical robustness of the results, with a significant reduction in analysis times. The FCM protocol developed in our laboratory [17] permitted us to demonstrate the capacity of (±)cis-4,4′-DMAR to statistically significantly increase the MNi frequency at 300 µM and the complete inability of (±)trans-4,4′-DMAR to do this.

The positive outcome obtained for (±)cis-4,4′-DMAR proves its capacity to induce structural and numerical chromosomal aberrations, and it stresses an additional alarming toxicological concern related to this NPS, also previously demonstrated for other stimulant drugs, such as some psychoactive phenethylamines [18] and the synthetic cathinone mexedrone [19]. On the other hand, the negative outcome for (±)trans-4,4′-DMAR demonstrates its inability to induce structural and numerical chromosomal aberrations, which is consistent with what has been demonstrated for other synthetic cathinones, i.e., α-PHP and α-PVP [19].

Our study does not allow us to hypothesize why (±)cis-4,4′-DMAR and (±)trans-4,4′-DMAR show different genotoxicities; nonetheless, the different outcomes confirm the well-known possibility to observe opposite biological effects in the cis- and trans- isomers of a compound, and it highlights the importance of testing the genotoxic potential, not only of a few representative molecules of an NPS class, but of every single NPS presenting even small differences in structure or conformation. 

In the present research, we co-treated TK6 cells with the non-genotoxic concentration 800 µM of (±)trans-4,4′-DMAR and different concentrations of (±)cis-4,4′-DMAR. The MNi frequency evaluation demonstrates that the association aggravated the genotoxic effect of (±)cis-4,4′-DMAR. In particular, it is very interesting that the treatment with (±)cis-4,4′-DMAR 200 µM alone did not induce a statistically significant MNi increase, while in association with (±)trans-4,4′-DMAR, it does. So, the co-treatments demonstrated that the assumption of both racemates aggravates the genotoxic effect. This worsening of 4,4′-DMAR toxicity agrees with an in vivo study that assessed a range of physiological and neuro-behavioural parameters in mice [15] and highlights another aspect to consider, i.e., that when the drug’s chemical structure presents multiple combinations of enantiomers and diastereomers, it is important to evaluate whether a possible mixture, when consumed, behaves as a single molecule, given the unpredictability of illicit drugs’ composition [3].

It bears noting that, although the MNvit makes it possible to evaluate whether the tested chemical is capable of inducing chromosomal aberrations and aneugenicity, “chemicals can induce genetic damage by different mechanisms, so a battery of tests sensitive to a different type of genetic damage are thought to provide the best assurance for detecting genotoxic hazard.” A specific approach includes a test to identify chromosomal aberrations and aneugenicity, for example, using the MNvit, and a bacterial reverse mutation test to identify point mutations [20]. Therefore, the outcome obtained in our study represents only the first but crucial step to hypothesize that (±)cis-4,4′-DMAR is mutagenic, while (±)trans-4,4′-DMAR is not. The next natural step is to proceed with further studies, performing a bacterial reverse-mutation test in order to confirm or completely exclude their mutagenic capacity, respectively. 

It is also important to consider that the present study was conducted in vitro; therefore, an in vivo corroboration could be advantageous. However, opinions about it are controversial. Indeed, “the limited sensitivity of in vivo tests in detecting a significant number of genotoxic compounds could be an argument against their use [21,22]. However, the concern that a small group of known or suspected human mutagens cannot be easily detected by in vitro tests could support the need for in vivo corroboration” [20,23].

Finally, for a more in-depth genotoxicological evaluation, the contribution of metabolites should be taken into account, also in consideration of the hypothesis formulated by Tirri et al.: “the urinary excretion studies suggested that the worsening of physiological and neuro-behavioural parameters could be related to the inhibition of the metabolism of the (±)cis-4,4′-DMAR form by the (±)trans-4,4′-DMAR“ [15].

Therefore, the studies will continue by re-testing the single racemates and their associations also in the presence of an exogenous source of metabolic activation as suggested by the OECD [24]. 

In conclusion, the present study encourages evaluating genotoxicity, too, as an additional alarming toxicological concern related to NPSs and advocates raising awareness not only of the acute effects of drugs of abuse, but also of the possibility of serious long-term consequences, given the key role played by genotoxicity in the development of numerous neuro- and chronic-degenerative diseases.

## 4. Materials and Methods

### 4.1. Reagents

Ethylenediaminetetraacetic acid (EDTA), Etoposide (ETP), Fetal Bovine Serum (FBS), L-Glutamine (L-GLU), Mitomycin C (MMC), Nonidet, Penicillin–Streptomycin solution (PS), Potassium Chloride, Potassium Dihydrogen Phosphate, Roswell Park Memorial Institute (RPMI) 1640 medium, Water bpc grade, Sodium Chloride, Sodium Hydrogen Phosphate, Vinblastine (VINB) (all purchased from Merck, Darmstadt, Germany), Guava Nexin Reagent (containing 7-aminoactinomycin (7-AAD) and Annexin-V-PE), Guava ViaCount Reagent (containing PI) (all purchased from Luminex Corporation, Austin, TX, USA), RNase A, SYTOX Green (purchased from Thermo Fisher Scientific, Waltham, MA, USA).

### 4.2. (±)cis-4,4′-DMAR and (±)trans-4,4′-DMAR

The (±)cis-4,4′-DMAR and (±)trans-4,4′-DMAR were synthetised and provided by the University of Ferrara Department of Organic Chemistry [15]. The test compounds were dissolved in absolute ethanol up to 80 mM stock solution and stored at –20 °C. Absolute ethanol concentration was always in the range 0–1% in all experimental conditions, to avoid potential solvent toxicity.

### 4.3. Cell Culture and Treatments

TK6 human lymphoblastoid cells were purchased by Merck (Darmstadt, Germany) and were grown at 37 °C and 5% CO_2_ in RPMI-1640 supplemented with 10% FBS, 1% L-GLU and 1% PS. To maintain exponential growth and considering that the time required to complete the cell cycle is 13–14 h, the cultures were divided every three days in fresh medium, and the cell density did not exceed the critical value of 9 × 10^5^ cells/mL.

In all the experiments, aliquots of 2.5 × 10^5^ of TK6 cells were treated with increasing concentrations of (±)cis-4,4′-DMAR or (±)trans-4,4′-DMAR or their association included in the range 0–800 µM and incubated for 26 h, corresponding to 1.5–2 replication cycles of the TK6 cells.

Cytotoxicity, apoptosis and MNi frequency evaluation were measured at the end of the 26 h treatment time.

### 4.4. Flow Cytometry

All FCM analyses reported below were performed using a Guava easyCyte 5HT flow cytometer equipped with a class IIIb laser operating at 488 nm (Luminex Corporation, Austin, TX, USA).

#### 4.4.1. Cytotoxicity Evaluation

Cytotoxicity assay was performed as previously described by Lenzi et al. [17,25] and Cocchi et al. [18]. Briefly, cells were stained with PI and 1000 cells per sample were analysed.

The viability percentage recorded in the treated cultures was normalized to that recorded in the concurrent negative control cultures, considered equal to 100%. These results confirmed that the cell viability percentage respected the OECD threshold (equal to 45 ± 5%) throughout all experimental conditions [16].

Meanwhile, always using PI reagent, the number of cells seeded at time zero and that measured at the end of the treatment time were evaluated to check the correct replication in the negative control cultures and compared to that measured in the treated cultures using RPD. Population Doubling (PD) and RPD were calculated with the following formulas:

(1) $$\mathrm{PD}=\log\left(\frac{\mathrm{post}-\mathrm{treatment}\;\mathrm{cell}\;\mathrm{number}}{\mathrm{initial}\;\mathrm{cell}\;\mathrm{number}}\right)÷\;\log2\;$$

(2) $$\mathrm{RPD}=\frac{\mathrm{PD}\;\mathrm{in}\;\mathrm{treated}\;\mathrm{cultures}}{\mathrm{PD}\;\mathrm{in}\;\mathrm{control}\;\mathrm{cutures}}\times 100$$

Similar to cytotoxicity, the cytostasis was checked in order to verify that cell proliferation respected the threshold established by the OECD guideline (equal to 45 ± 5%) [16].

#### 4.4.2. Apoptosis Evaluation

The percentage of apoptotic cells was evaluated according to the procedure used by Lenzi et al. [25].

Briefly, the percentage of apoptotic cells was assessed by means of a double-staining protocol with 7-AAD and Annexin-V-PE and analysing 2000 cells per sample.

The apoptotic cell percentage recorded in the treated cultures was normalized to that recorded in the concurrent negative cultures, considered equal to 1, and expressed as apoptotic fold increase. These results were used to check that the apoptosis induction was similar or corresponding at most to a doubling of that recorded in the concurrent negative cultures. A concentration of 5 µg/mL of ETP was used as positive control.

#### 4.4.3. MNi Frequency Evaluation

The analysis of the MNi frequency was performed using an automated protocol published by Lenzi et al. [17]. Briefly, at the end of the treatment time, cells were collected, lysed and stained with SYTOX Green. The discrimination between nuclei and MNi was performed based on the different sizes analysed by Forward Scatter (FSC) and the different intensities of green fluorescence. 

The MNi frequency, calculated as the number of MNi per 10,000 nuclei deriving from living and proliferating cells for every sample and recorded in treated cultures at all the concentrations tested, was normalized to those recorded in the concurrent negative control cultures.

We used the clastogen MMC and the aneugen VINB as positive controls [16].

### 4.5. Statistical Analysis

Each test chemical concentration was tested in triplicate at all the experimental conditions. All analyses were repeated three times. RPD, viability percentage, apoptosis fold increase and MNi frequency fold increase were expressed as mean ± SEM. At all experimental conditions, more than three groups of matched data were compared, so statistical significance was analysed by one-way repeated measures ANOVA, followed by Dunnett or Bonferroni as post-tests to compare all treated groups with the control group. We considered the difference between means statistically significant if *p* value < 0.05.

We used Prism Software 4.

## Figures and Tables

**Table 1:** RPD of TK6 cells after 26 h treatment with (±)cis-4,4′-DMAR or (±)trans-4,4′-DMAR at the indicated concentrations compared to the concurrent negative control [0 µM]. Each value represents the mean ± SEM of three independent experiments. Data were analysed using one-way repeated measures ANOVA followed by Dunnet as post-test. ** *p* < 0.01 vs. [0 µM]; *** *p* < 0.001 vs. [0 µM]. The red line separates the RPDs complying or not with the OECD threshold for cellular replication (45 ± 5%).

Concentration	RPD (±)cis-4,4′-DMAR	RPD (±)trans-4,4′-DMAR
0 µM	100.0%	100.0%
25 µM	91.5 ± 4.2%	99.2 ± 0.8%
50 µM	90.6± 3.3%	99.1 ± 0.4%
100 µM	92.9 ± 3.5%	98.9 ± 0.3%
200 µM	84.9 ± 2.2% **	94.0 ± 3.8%
300 µM	58.2 ± 5.7% ***	97.8 ± 2.2%
400 µM	18.9 ± 2.2% ***	98.3 ± 1.7%
800 µM	/	99.0 ± 1.0%

**Table 2:** RPD of TK6 cells after 26 h co-treatment with (±)cis-4,4′-DMAR and (±)trans-4,4′-DMAR at the indicated concentrations compared to the concurrent negative control [0 µM]. Each value represents the mean ± SEM of three independent experiments. Data were analysed using one-way repeated measures ANOVA followed by Bonferroni as post-test. * *p* < 0.05 vs. [0 µM].

Concentration	RPD
0 µM	100.0%
cis 100 µM + trans 800 µM	99.5 ± 0.5%
cis 200 µM + trans 800 µM	90.7± 5.3%
cis 300 µM + trans 800 µM	45.4 ± 1.2% *

## Data Availability

Not applicable.
